# Metabolic biomarkers in early detection of gestational diabetes mellitus: a prospective diagnostic accuracy study

**DOI:** 10.3389/fmed.2026.1765602

**Published:** 2026-01-15

**Authors:** Alihan Tigli, Yakup Baykus, Rulin Deniz, Kevser Tari Selcuk, Nazli Sener, Yasemin Ercan Degirmenci, Guzide Ece Akinci, İlknur Zeynep Acarturk, Nurseda Sertdemir, Suleyman Aydin

**Affiliations:** 1 Department of Obstetrics and Gynecology, Faculty of Medicine, Bandırma OnYedi Eylül University, Bandırma, Türkiye; 2 Department of Nutrition and Dietetics, Faculty of Health Sciences, Bandırma OnYedi Eylül University, Bandırma, Türkiye; 3 Bandırma Training and Research Hospital Gynecology and Obstetrics Clinic, Bandırma, Türkiye; 4 Department of Internal Medicine, Health Ministration Gazi State Hospital, Samsun, Türkiye; 5 Department of Medical Biochemistry, Faculty of Medicine, Fırat University, Elazig, Türkiye

**Keywords:** atherogenic index plasma, early diagnosis, gestational diabetes mellitus, HDL-based ratios, inflammatory markers, triglyceride-glucose index

## Abstract

**Background and objectives:**

Early diagnosis of gestational diabetes mellitus (GDM) is essential for the prevention of maternal and neonatal complications. In this study, we aimed to investigate the value of first trimester inflammatory markers (Neutrophil-to-Lymphocyte Ratio (NLR), Platelet-to-Lymphocyte Ratio (PLR), Monocyte-to-Lymphocyte Ratio (MLR), Neutrophil-to-Monocyte Ratio (NMR)), HDL-based metabolic inflammation indicators (Lymphocyte-to-High-Density Lipoprotein Cholesterol Ratio (LHR), Monocyte-to-High-Density Lipoprotein Cholesterol Ratio (MHR), Granulocyte-to-High-Density Lipoprotein Cholesterol Ratio (GHR)) and metabolic indices (Atherogenic Index of Plasma (AIP), Triglyceride–Glucose Index (TyG)) in predicting GDM.

**Materials and methods:**

Between December 2024 and October 2025, 250 pregnant women were included in this prospective diagnostic accuracy study conducted at Bandırma Onyedi Eylül University Hospital. First trimester (10–14 weeks) complete blood count, fasting glucose and lipid profile were recorded. NLR, PLR, MLR, NMR, LHR, MHR, GHR, AIP and TyG indices were calculated. GDM was diagnosed with 75 g Oral Glucose Tolerance Test (OGTT) at 24–28 weeks according to International Association of the Diabetes and Pregnancy Study Groups (IADPSG) criteria. Diagnostic performance was evaluated by ROC analysis and significance level was taken as *p* < 0.05.

**Results:**

The prevalence of GDM was 18.0% (*n* = 45). Age, body weight, Body Mass Index (BMI) and parity were significantly higher in the GDM group (*p* < 0.05). Biomarker averages did not differ significantly between the two groups (*p* > 0.05). According to Receiver Operating Characteristic (ROC) analysis, the Area Under the Curve (AUC) values were NLR (0.470), PLR (0.490), MLR (0.474), NMR (0.481), LHR (0.567), MHR (0.506), GHR (0.466), AIP (0.561) and TyG (0.573). None of them exceeded the threshold value of 0.70.

**Conclusion:**

LHR, MHR, AIP and TyG indices are of limited value in predicting GDM. These markers cannot be used alone in the early period. OGTT is still the most reliable diagnostic method. More specific biomarkers and multicenter studies are needed for early diagnosis.

## 1 Introduction

Gestational diabetes mellitus (GDM) is defined by the International Diabetes Federation (IDF) as “a condition that begins during pregnancy or is first recognised during pregnancy, in which blood sugar levels are higher than normal but not as high as in overt diabetes.” GDM, is one of the most common complications of pregnancy, affecting approximately 16 per cent of pregnancies worldwide (1, 2).

It is a major cause of both maternal and neonatal morbidity and mortality. Maternal complications include increased risk of type 2 diabetes mellitus and cardiovascular disease, preterm labour, polyhydramnios, pre-eclampsia and the need for elective caesarean section (3, 4), neonatal complications, including neonatal intensive care unit admission, hypoglycaemia, neonatal jaundice, macrosomia, shoulder dystocia and respiratory distress syndrome, and disorders of glucose metabolism, including obesity, insulin resistance, reduced acute insulin response and impaired glucose tolerance (3, 5, 6).

According to current guidelines, the clinical diagnosis of GDM is made with a 75 g Oral Glucose Tolerance Test (OGTT) at 24–28 weeks of gestation (7–9). However, current evidence suggests that up to this gestational week, both mother and foetus may be adversely affected to varying degrees by maternal hyperglycaemia (10–12). It has also been reported that treatment of GDM initiated before 20 weeks of gestation may reduce adverse maternal and neonatal outcomes (13). Therefore, detection of GDM in the early gestational period is critical for the prevention of possible complications (14). However, the most important problem in the early diagnosis of GDM is that the optimal screening tool and diagnostic thresholds have not yet been standardised (15). In the literature, it is emphasised that the diagnostic accuracy of first or second trimester maternal biomarkers may be important for the early diagnosis of GDM and some biomarkers have been reported to give promising results in GDM prediction (14, 16, 17). The pathophysiology of GDM is a multifactorial and complex process characterised by the interaction of complex factors such as increased insulin resistance, beta-cell dysfunction and dynamic changes in maternal inflammatory and metabolic response (18). These factors have a synergistic effect in the development of GDM. The increase in placental hormones (human placental lactogen, progesterone, cortisol, and prolactin) during pregnancy leads to significant changes in maternal metabolism. These hormones contribute to the development of physiological insulin resistance, especially in the second and third trimesters of pregnancy. In normal pregnancy, pancreatic *β*-cells meet the increased insulin demand by compensatory insulin secretion, but this compensation mechanism is insufficient in GDM. This multifactorial nature of GDM pathophysiology requires the evaluation of multiple biomarkers reflecting different metabolic and inflammatory pathways for early diagnosis of the disease.

In recent years, the hypothesis that increased inflammatory responses during pregnancy play a central role in the development of GDM has become the focus of research. Abnormal adaptation of the maternal immune system to pregnancy predisposes to the development of chronic moderate inflammation. Increased levels of proinflammatory cytokines such as TNF-*α*, IL-6, IL-1β and C-reactive protein contribute to the development of insulin resistance by impairing insulin signalling. It has been reported that pregnancies with GDM show an increase in oxidative stress and inflammation markers and changes in antioxidant defence mechanisms in circulation, placenta, adipose tissue and skeletal muscle compared to pregnancies with normal glucose tolerance (19, 20). Based on this pathophysiological basis, it has been suggested that easily calculable inflammatory markers, such as the neutrophil-to-lymphocyte ratio (NLR), platelet-to-lymphocyte ratio (PLR), monocyte-to-lymphocyte ratio (MLR), and neutrophil-to-monocyte ratio (NMR), may help predict GDM. These parameters are advantageous in clinical practice because they can be calculated with values obtained from a complete blood count, are non-invasive, low cost and provide rapid results. These ratios, which reflect the degree of systemic inflammation, are thought to be able to detect the inflammatory component of GDM at an early stage.

Metabolic inflammation and lipid metabolism disorders are other important components of GDM pathogenesis. Recent studies have revealed that high-density lipoprotein cholesterol (HDL-C) has strong anti-inflammatory, antioxidant and antithrombotic properties beyond its role in cholesterol transport (21, 22). These properties make HDL an important marker in the context of metabolic inflammation (23). It has been reported that the imbalance between the impairment of these protective functions of HDL and the increase in the inflammatory response in GDM may play a critical role in the development of the disease. In this context, it has been suggested that new parameters calculated by the ratio of immune cell numbers to HDL-C can be used to predict metabolic inflammatory disorders. These new indices, such as lymphocyte/HDL-C ratio (LHR), monocyte/HDL-C ratio (MHR) and granulocyte/HDL-C ratio (GHR), provide an indicator that combines both the intensity of the inflammatory response (via immune cell counts) and the potential of HDL to stabilise this inflammation. These HDL-based indicators of metabolic inflammation provide a more holistic perspective on the pathophysiology of GDM, allowing both inflammatory and metabolic components of the disease to be assessed together.

Insulin resistance and dyslipidaemia constitute the cornerstones of GDM pathophysiology. In the later weeks of pregnancy, as physiological insulin resistance reaches pathological levels, glucose uptake by peripheral tissues decreases and hepatic glucose production increases in parallel. These metabolic changes not only disrupt glucose homeostasis but also lead to marked disturbances in lipid metabolism. In this context, atherogenic lipid profile features such as increased triglyceride levels, decreased HDL-cholesterol levels and formation of small dense Low-Density Lipoprotein (LDL) particles occur.

In order to assess these lipid metabolism disorders, the atherogenic index plasma (AIP) has emerged as an innovative marker. AIP is calculated as the logarithm of the ratio of triglyceride (TG) and HDL-C [log10 (TG/HDL-C)] and was first described by Dobiášová and Frohlich in 2001 as an indicator of atherosclerosis risk (24). Studies have reported that AIP is associated with type 2 diabetes mellitus, prediabetes, metabolic syndrome and cardiovascular diseases. The AIP value, which reflects high TG and low HDL-C ratio, is also valuable in terms of showing the presence of small dense LDL particles, atherogenic lipid profile and the degree of visceral adiposity. The pathophysiological basis of the relationship between AIP and GDM has not yet been fully elucidated. Mechanisms include AIP triggering chronic moderate inflammation, increasing oxidative stress and causing endothelial dysfunction. Furthermore, AIP-associated lipid profile alterations exacerbate insulin resistance by increasing the release of free fatty acids, thereby contributing to impaired glucose homeostasis. Given these multifaceted pathophysiological interactions, some investigators have suggested that AIP may be a reliable predictor of GDM in pregnancy (25). Similarly, a metabolic indicator used to assess insulin resistance is the triglyceride-glucose (TyG) index, which is calculated based on fasting blood glucose and fasting TG levels. The TyG index is calculated by the formula ln [fasting triglyceride (mg/dL) × fasting glucose (mg/dL)/2 (26, 27). In the literature, it has been shown that TyG index may be associated with various metabolic disorders such as type 2 diabetes mellitus, metabolic syndrome, cardiovascular diseases and non-alcoholic fatty liver disease (28, 29). Considering that insulin resistance is one of the main pathophysiologies of GDM, it has been suggested that TyG index may be a potential marker for predicting GDM. Metabolic indices such as AIP and TyG index may offer the opportunity to evaluate the metabolic pathophysiology of the disease at an early stage by reflecting the insulin resistance and lipid metabolism disorder components of GDM.

Although the existing literature shows that various biomarkers have been investigated for the early diagnosis of GDM, comprehensive studies evaluating inflammatory markers, HDL-based metabolic inflammation indicators and metabolic indices together are limited. Considering the multifactorial pathophysiology of GDM, the evaluation of multiple parameters reflecting the three main pathophysiological components of the disease (inflammation, metabolic inflammation and insulin resistance/lipid metabolism disorder) may offer a more powerful and holistic approach in the early diagnosis of the disease. In this context, systematic investigation of the role of easily available, cost-effective and non-invasive parameters in the early prediction of GDM may provide important contributions to clinical practice.

The aim of this diagnostic accuracy study was to examine the early predictive value of biomarkers reflecting the three main pathophysiological mechanisms of GDM. In this context, the ability of nine parameters including (1) inflammatory markers (NLR, PLR, MLR, and NMR), (2) HDL-based metabolic inflammation indicators (LHR, MHR, GHR) and (3) metabolic indices (AIP and TyG index) to detect and predict GDM in early pregnancy was evaluated. Our study aims to determine whether the combined evaluation of these three different categories of biomarkers based on the multifactorial pathophysiology of GDM can provide a clinically usable approach for the early diagnosis of the disease.

## 2 Materials and methods

### 2.1 Study design and sampling

This prospective diagnostic accuracy study was conducted with pregnant women who applied to Bandırma Onyedi Eylül University Hospital Obstetrics and Gynaecology Outpatient Clinic between December 2024 and October 2025. The minimum sample size required for the study was calculated as 207 using the OpenEpi 3.01 programme, based on the GDM prevalence (16%) reported by Aydın et al., with a 5% margin of error and 95% confidence interval (30). However, given the possibility of data loss and missing information, the sample size was increased by 20%, and the study was planned to include at least 248 pregnant women.

Pregnant women who presented to our hospital between 10 and 14 weeks of gestation between the specified dates were evaluated for the study. A total of 810 pregnant women were interviewed during this period. As a result of the initial evaluations, a total of 252 pregnant women were excluded from the study because 11 pregnant women presented due to emergency, 12 pregnant women were not in the 18–40 age group, 18 pregnant women had multiple pregnancies, 36 pregnant women had a diagnosis of pregestational diabetes, 89 pregnant women had chronic diseases (hypertension, cardiovascular diseases, renal diseases, thyroid diseases), 24 pregnant women had active infections, 18 pregnant women were using immunosuppressive therapy or corticosteroids, and 44 pregnant women did not have complete blood count and biochemical examinations at 10–14 weeks of gestation. A total of 252 pregnant women were excluded from the study because they did not have complete blood count and biochemical examinations performed at 10–14 weeks of gestation.

**Figure 1:**
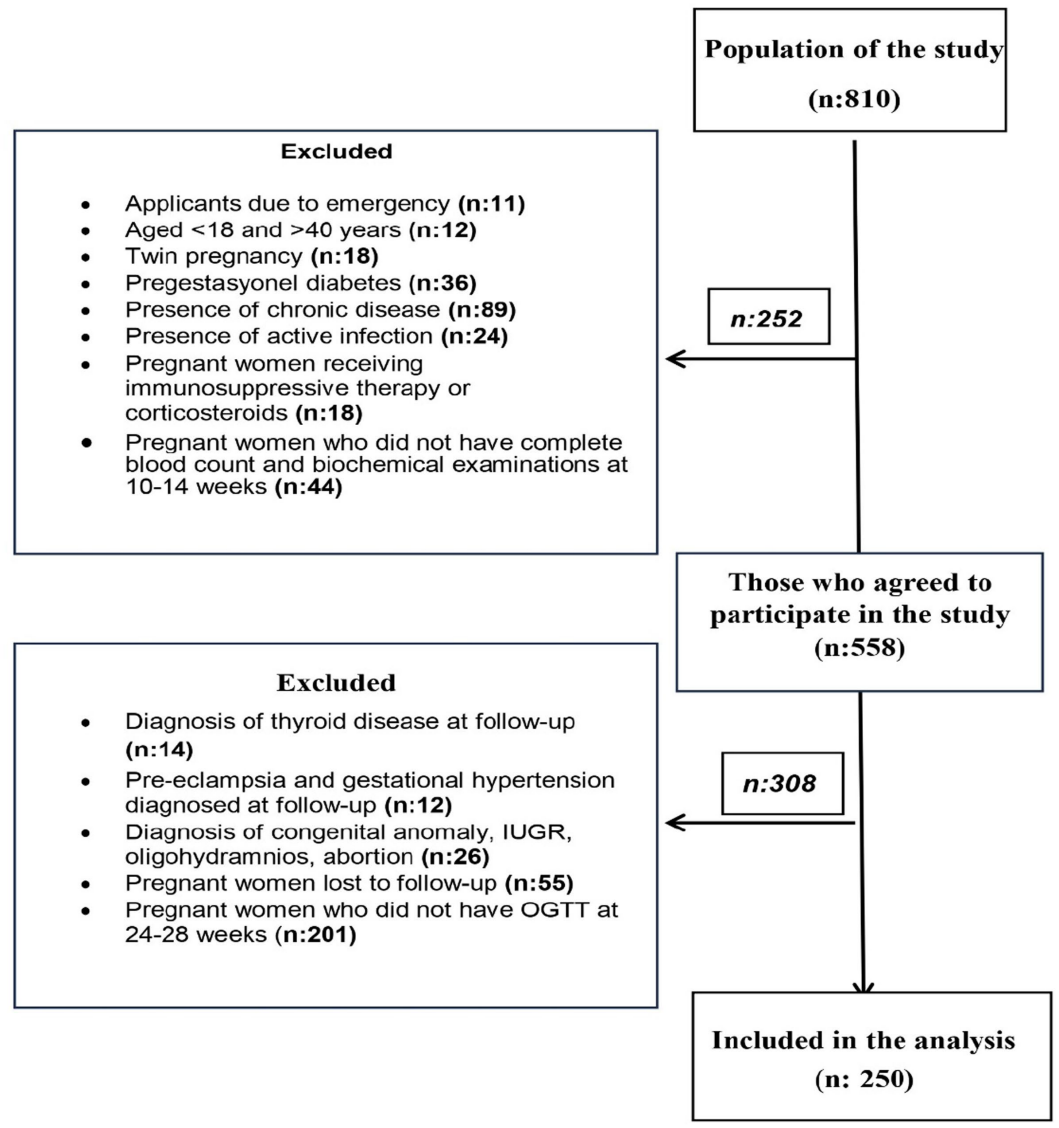
Flowchart of participant inclusion in the study.

Pregnant women included in the study were followed up and evaluated for 75 g OGTT for the second time at 24–28 weeks of gestation. A total of 308 pregnant women were excluded from the study, including 14 pregnant women with a diagnosis of thyroid disease, 12 pregnant women with a diagnosis of pre-eclampsia and gestational hypertension, 26 pregnant women with poor obsteric results (congenital anomaly, intrauterine growth restriction (IUGR), oligohydramnios, abortion), 55 pregnant women who did not come for follow-up at 24–28 weeks and 201 pregnant women who did not have OGTT test. As a result, the data obtained from a total of 250 pregnant women who voluntarily participated in the study were analysed (Figure 1).

### 2.2 Data collection tools

In the study, “Descriptive Information Form” and “Data Collection Form” prepared by the researchers by reviewing the literature were used as data collection tools.

The descriptive information form consisted of questions about the age, education level, smoking status during pregnancy, physical activity status, family history of diabetes, gestational week, number of pregnancies, and number of living children.

Laboratory data (complete blood count showing neutrophil, lymphocyte, platelet, monocyte counts, fasting blood glucose, TG, HDL-C and 75 g OGTT values) were obtained from hospital records and added to the data collection form.

Complete blood count (haemoglobin, haematocrit, white blood cell count, neutrophil count, lymphocyte count, neutrophil count, lymphocyte count, monocyte count, platelet count), biochemical test results (fasting blood glucose, total cholesterol), TG, HDL-C and 75 g OGTT results (fasting, 1st hour and 2nd hour glucose values) performed at 10–14 weeks of gestation of the participants included in the study were obtained from hospital records and added to the data collection form and recorded.

### 2.3 Data collection method

Pregnant women who applied to our hospital were informed about the purpose and scope of the study. Pregnant women who agreed to participate in the study and were accepted to the study according to the inclusion and exclusion criteria were asked to sign the informed consent form. “Introductory Information Form” and “Data Collection Form” were applied by the researchers to the pregnant women who signed the informed consent form. Then, the body weight and height of the pregnant women were measured by standard procedures and Body Mass Index (BMI) was calculated by dividing the body weight by the square of the height30. For each pregnant woman included in the study, the results of complete blood count including neutrophil, lymphocyte, platelet, monocyte counts, fasting blood glucose, TG, HDL-C measurements performed at 10–14 weeks of pregnancy, and the results of 75 g OGTT test performed at 24–28 weeks were recorded on the data collection form.

### 2.4 Laboratory analyses

Venous blood samples were collected in the morning hours at 10–14 weeks of gestation following an overnight fast of at least 8 h. All participants were instructed to refrain from eating or drinking anything except water after their evening meal the night before the blood draw. Complete blood count was performed with an automatic haematology analyser (Sysmex XN-1000, Sysmex Corporation, Kobe, Japan) from blood samples collected in ethylenediaminetetraacetic acid (EDTA) tubes. Biochemical parameters (glucose, total cholesterol, TG, HDL-C) were measured by enzymatic colorimetric method in serum obtained after centrifugation from blood samples collected in gel biochemistry tubes using an automatic biochemistry analyser (Cobas 6,000, Roche Diagnostics, Mannheim, Germany). All analyses were performed following daily quality control procedures.

The diagnosis of GDM was made using International Association of the Diabetes and Pregnancy Study Groups (IADPSG) criteria according to the results of 75 g OGTT performed at 24–28 weeks of gestation. According to IADPSG criteria, fasting plasma glucose ≥92 mg/dL, 1st hour plasma glucose ≥180 mg/dL or 2nd hour plasma glucose ≥153 mg/dL is sufficient for the diagnosis of GDM (31). Pregnant women fasted for at least 8 h before the OGTT and were kept in a sitting position during the test.

Then, for each pregnant woman included in the study, NLR, PLR, MLR, NMR values using neutrophil, lymphocyte, platelet, monocyte counts, AIP using TG and HDL-C values, TyG index using fasting blood glucose and TG levels, Lymphocyte/HDL-C, Monocyte/HDL-C, Granulocyte/HDL-C values using lymphocyte, monocyte, granulocyte and HDL-C values were calculated with the relevant formulas as stated in the literature (14, 16, 17, 32–36).

### 2.5 Data analysis

SPSS 22.0 package programme was used to evaluate the data. Number, percentage, mean and standard deviation values were calculated in data analysis. The suitability of the variables for normal distribution was evaluated using skewness and kurtosis coefficients, with values between −1 and +1 considered acceptable. Continuous variables were expressed as mean ± standard deviation (SD). For intergroup comparisons between pregnant women with and without GDM, the Student’s t-test was used for normally distributed variables, and the Mann–Whitney U test was applied for non-normally distributed parameters. Binary logistic regression analyses were performed to evaluate the associations between first-trimester biochemical and immunological parameters and the presence of GDM. The variables included in the adjusted models were selected based on their established roles as risk factors for GDM in the literature, their potential confounding effects on the relationship between metabolic biomarkers and GDM, and their significant associations with GDM in univariate analyses. Accordingly, maternal age, pre-pregnancy BMI, family history of diabetes, and number of pregnancies were included in the models as potential confounders. Three hierarchical models were constructed: Model 1 was the crude model including only biochemical and immunological parameters. Model 2 was adjusted for maternal age (continuous) and pre-pregnancy BMI (continuous). Model 3 was further adjusted for maternal age (continuous), pre-pregnancy BMI (continuous), family history of diabetes (no = 0, yes = 1), and number of pregnancies (1–2 = 0, ≥3 = 1). Odds ratios (ORs) with 95% confidence intervals (CIs) were calculated for all models. Receiver Operating Characteristic (ROC) curve analysis was performed to determine the discriminative power of biomarkers in the diagnosis of GDM. Area under Curve (AUC), Sensitivity, Specificity, Positive Likelihood Ratio (LR+) and Negative Likelihood Ratio (LR-) were calculated for each biomarker. AUC values between 0.9–1.0 were interpreted as excellent, 0.8–0.9 as very good, 0.7–0.8 as good, 0.6–0.7 as moderate and 0.5–0.6 as poor discriminative power (37–39). Youden index was calculated to determine the optimal cut-off points of biomarkers for the diagnosis of GDM and the highest index value was determined as the cut-off point. A post-hoc power analysis was performed based on the observed GDM prevalence (18%) and the total sample size (*n* = 250). At a significance level of 0.05, the study had approximately 88% power to detect an odds ratio (OR) of 2.0, while the power decreased to 71% for an OR of 1.7 and to 52% for an OR of 1.5. The significance level of statistical tests was accepted as *p* < 0.05.

## 3 Results

### 3.1 Sociodemographic and clinical characteristics of participants

The mean age of the 250 pregnant women included in our study was 28.17 ± 4.93 years. It was observed that the mean age was statistically significantly higher in the group diagnosed with GDM compared to the control group (*p* < 0.05). Analysis of the participants’ educational profiles revealed that approximately half (42.8%) had university or postgraduate education. While the prevalence of smoking was found to be 21.6%, almost half of the pregnant women (49.2%) reported regular physical activity. As a remarkable finding, 40.4% of the participants had a history of diabetes in first-degree relatives.

When obstetric parameters were evaluated, mean gestational age was 25.42 ± 1.56 weeks. The rate of primigravidae was 49.2% and the rate of nulliparous pregnancies was 54.4%. In the parity comparison, the rate of having three or more children was significantly higher in the GDM group than in the control group (*p* < 0.05).

### 3.2 Anthropometric findings and metabolic parameters

Anthropometric measurements revealed that the mean body weight of pregnant women was 75.74 ± 14,56 kg, height 162.66 ± 6,42 cm and BMI: 28.61 ± 5,12 kg/m^2^. In the intergroup comparison, body weight and BMI values of GDM positive pregnant women were found to be significantly higher (*p* < 0.05) and height was found to be lower (p < 0.05). According to 75 g OGTT results, the mean plasma glucose level at the 1st hour was 136.34 ± 36,72 mg/dL and at the 2nd hour was 110.61 ± 31,84 mg/dL. At both time points, glucose values of the GDM group were significantly higher than those of the control group (*p* < 0.05; Table 1).

**Table 1:** Comparison of clinical, demographic, and laboratory characteristics between GDM and control groups.

Descriptive characteristics	All groups (*n* = 250)	GDM (+) (*n* = 45)	GDM (−) (*n* = 205)	*p*
	% (*n*) Mean ± SD	% (*n*) Mean ± SD	% (*n*) Mean ± SD	
Age (years)	28,17 ± 4.93	30.93 ± 5.27	27.57 ± 4.65	<0.001^*^
Education level				
Secondary school and below	24.4 (61)	35.6 (16)	22.0 (45)	0.103^**^
High school	32.8 (82)	33.3 (15)	32.7 (67)	
University and above	42.8 (107)	31.1 (14)	45.4 (93)	
Smoking in pregnancy				
Current smoker	21.6 (54)	15.6 (7)	22.9 (47)	0.277^**^
Non-smoker	78.4 (196)	84.4 (38)	77.1 (158)	
Physical activity				
Yes	49.2 (123)	422.2(19)	50.7 (104)	0.301^**^
No	50.8 (127)	57.8(26)	49.3 (101)	
Family history of diabetes				
Yes	40.4 (101)	53.3 (24)	37.6 (77)	0.051^**^
No	59.6 (149)	46.7 (21)	62.4 (128)	
Gestational age (weeks)	25.42 ± 1.56	25.47 ± 1.57	25.41 ± 1.55	0.825^*^
Number of pregnancies				
1	49.2(123)	33.3(15)	52.7(108)	0.008^**^
2	26.0(65)	24.4(11)	26.3(54)	
≥3	24.8(62)	42.3(19)	21.0(43)	
Number of living children				
0	54.4 (136)	42.2 (19)	57.1 (117)	0.054^**^
1	31.6 (79)	33.3 (15)	31.2 (64)	
≥2	14.0 (35)	24.4 (11)	11.7 (24)	
Anthropometric measurement				
Weight (kg)	75.74 ± 14.56	80.47 ± 13.31	74.70 ± 14.64	0.016^*^
Height (cm)	162.66 ± 6.42	160.96 ± 6.36	163.04 ± 6.38	0.049^*^
BMI (kg/m^2^)	28.61 ± 5.12	31.09 ± 4.88	28.06 ± 5.02	<0.001^*^
OGTT fasting, mg/dL				
OGTT 1-h, mg/dL	136.34 ± 36.72	188,81 ± 29.52	125.08 ± 27.10	<0.001^*^
OGTT 2-h, mg/dL	110.61 ± 31.84	155.79 ± 28.22	100.90 ± 22.99	<0.001^*^

SD, Standard Deviation; GDM, Gestational Diabetes Mellitus; BMI, Body Mass Index; *Student’s *t* test. **Pearson Chi square test. Bold values indicate statistically significant *p* Value.

### 3.3 GDM frequency

**Figure 2:**
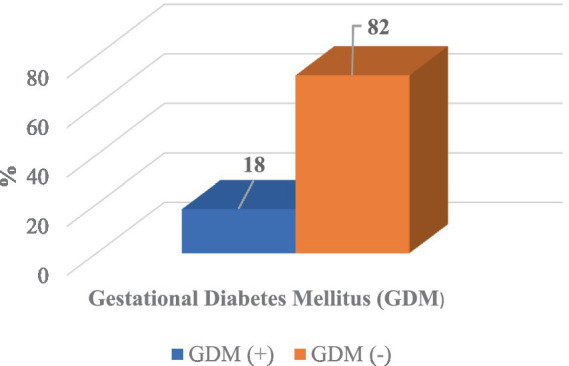
Prevalence of gestational diabetes mellitus.

The prevalence of GDM in our cohort was 18.0% (Figure 2), which is consistent with current literature rates.

### 3.4 Inflammatory and lipid-based biomarkers

The inflammatory and lipid-based indices evaluated in our study are presented in detail in Table 2.

**Table 2:** Comparison of inflammatory and metabolic biomarkers in pregnant women with and without GDM.

Parameters	All groups (*n* = 250)	GDM (+) (*n* = 45)	GDM (−) (*n* = 205)	*p*
	Mean ± SD	Mean ± SD	Mean ± SD	
NLR	3.12 ± 1.15	2.97 ± 1.07	3.16 ± 1.170	0.527^*^
PLR	129.79 ± 38.51	129.14 ± 37.63	129.94 ± 38.79	0.829^*^
MLR	0.74 ± 7.10	0.28 ± 0.07	0.84 ± 7.92	0.583^*^
NMR	10.91 ± 3.12	10.72 ± 2.93	10.95 ± 3.17	0.649^**^
Lymphocyte/HDL-C	0.03 ± 0.01	0.04 ± 0.01	0.03 ± 0.01	0.189^**^
Monocyte/HDL-C	0.02 ± 0.27	0.01 ± 0.00	0.03 ± 0.31	0.907^*^
Granulocyte/HDL-C	0.11 ± 0.04	0.12 ± 0.05	0.12 ± 0.04	0.949^**^
AIP	0.27 ± 0.22	0.32 ± 0.23	0.27 ± 0.21	0.161^**^
TyG Index	8.47 ± 0.49	8.60 ± 0.52	8.44 ± 0.48	0.069^**^

SD, Standard Deviation; GDM, Gestational Diabetes Mellitus; NLR, Neutrophil Lymphocyte Ratio; PLR, Platelet-Lymphocyte Ratio; MLR, Monocyte Lymphocyte Ratio; NMR, Neutrophil Monocyte Ratio; Lymphocytes/HDL-C, Lymphocyte High Density Lipoprotein Ratio; Monocytes/HDL-C, Monocyte High Density Lipoprotein Ratio; Granulocytes/HDL-C, Granulocyte High Density Lipoprotein Ratio; AIP, Plasma Atherogenic Index; TyG Index, Triglyceride Glucose Index. *Mann Whitney U test. **Student’s *t* test.

When the mean values calculated for all participants were analysed; NLR 3.12 ± 1.15, PLR 129.79 ± 38.51, MLR 0.74 ± 7.1, NMR 10.91 ± 3.12.

Lipid-based ratios were determined as follows: LHR: 0.03 ± 0.01, MHR: 0.02 ± 0.27, GHR: 0.11 ± 0.04. In addition, AIP: 0.27 ± 0.22 and TyG index: 8,47 ± 0,49 were calculated.

No statistically significant association was found between GDM and these inflammatory and lipid-based biomarkers (*p* > 0.05; Table 2).

### 3.5 Association of first-trimester biochemical and immunological parameters with GDM

The results of the binary logistic regression analyses are presented in Table 3.

**Table 3:** Binary logistic regression analysis evaluating the independent associations of first-trimester biochemical and immunological parameters with GDM.

Parameters	Model 1 (Crude Model)		Model 2 (Adjusted Model)		Model 3 (Adjusted Model)	
	UOR (95% CI)	*p*	UOR (95% CI)	*p*	UOR (95% CI)	*p*
NLR	0.858 (0.634; 1.161)	0.321	0.836 (0.603; 1.161)	0.286	0.823 (0.589; 1.149)	0.252
PLR	0.999 (0.991; 1.008)	0.900	0.999 (0.989; 1.008)	0.755	0.999 (0.989; 1.008)	0.750
MLR	0.185 (0.004; 7.757)	0.376	0.342 (0.007; 16.697)	0.588	0.300 (0.006; 15.009)	0.546
NMR	0.976 (0.879; 1.083)	0.647	0.941 (0.838; 1.056)	0.302	0.940 (0.839; 1.054)	0.291
Lymphocyte/HDL-C	2.663 (0.581; 9; 617)	0.191	2.486 (0.493; 8.517)	0.306	2.311 (0.418; 7.596)	0.239
Monocyte/HDL-C	0.333 (0.104; 1.090)	0.835	0.317 (0.096; 1.002)	0.834	0.286(0.082;0,994)	0.805
Granulocyte/HDL-C	1.301(0.011; 6.640)	0.949	0.965 (0.040; 5.307)	0.828	0.940 (0.036; 5.235)	0.924
AIP	2.967 (0.647; 13.601)	0.162	1.327 (0.250; 7.028)	0.740	1.176 (0.216; 6.399)	0.852
TyG Index	1.888 (0.949; 3.756)	0.070	1.169 (0.551; 2.482)	0.685	1.086 (0.502; 2.352)	0.834

NLR, Neutrophil Lymphocyte Ratio; PLR, Platelet-Lymphocyte Ratio; MLR, Monocyte Lymphocyte Ratio; NMR, Neutrophil Monocyte Ratio; Lymphocytes/HDL-C, Lymphocyte High Density Lipoprotein Ratio; Monocytes/HDL-C, Monocyte High Density Lipoprotein Ratio; Granulocytes/HDL-C, Granulocyte High Density Lipoprotein Ratio; AIP, Plasma Atherogenic Index; TyG Index, Triglyceride Glucose Index. Model 1: Crude model. Model 2. Adjusted for maternal age (continuous), pre-pregnancy BMI (continuous). Model 3. Adjusted for maternal age (continuous), pre-pregnancy BMI (continuous), Family history of diabetes (No = 0, Yes = 1), and Number of pregnancies (1–2 = 0, ≥3 = 1).

In the crude models, none of the first-trimester biochemical or immunological parameters were significantly associated with GDM. After adjustment for maternal age and pre-pregnancy BMI, and after further adjustment for family history of diabetes and number of pregnancies, none of the evaluated parameters were significantly associated with GDM (all *p* > 0.05).

### 3.6 Predictive value analysis

**Figure 3:**
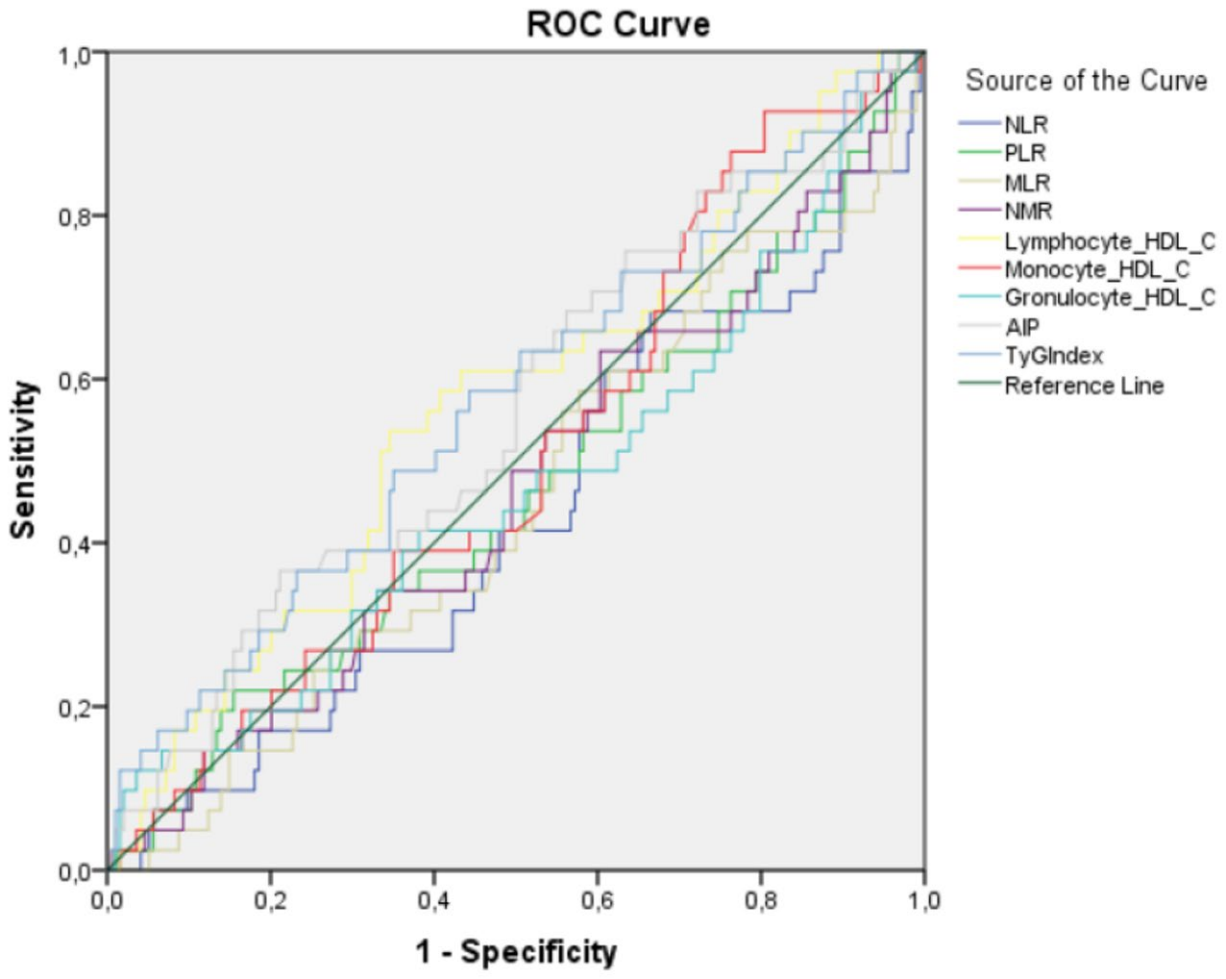
ROC curves of biochemical and immunological parameters in determining gestational diabetes.

The diagnostic performance of the biomarkers evaluated by ROC curve analysis for GDM prediction is shown in Table 4 and Figure 3.

**Table 4:** Diagnostic performance of biochemical and immunological parameters for the prediction of GDM.

Parameters	AUC (95% CI)	SE	*p*	Cut of value	Sensitivity (%)	Specificity (%)	LR(+) (%)	LR(−) (%)	Youden index
NLR	0.470 (0.374;0.566)	0.049	0.528	2.522	0.711	0.356	1.104	0.811	0.067
PLR	0.490 (0.392;0.587)	0.050	0.829	162.741	0.244	0.854	1.670	0.885	0.098
MLR	0.474 (0.380;0.568)	0.048	0.585	0.229	0.778	0.263	1.055	0.843	0.041
NMR	0.481 (0.387;0.575)	0.048	0.691	9.720	0.667	0.400	1.111	0.833	0.067
Lymphocyte/HDL-C	0.567 (0.468;0.665)	0.050	0.181	0.041	0.537	0.655	1.553	0.707	0.191
Monocyte/HDL-C	0.506 (0.412;0.600)	0.048	0.909	0.006	0.927	0.196	1.152	0.373	0.123
Granulocyte/HDL-C	0.466 (0.362;0.570)	0.053	0.496	0.200	0.122	0.964	3.379	0.911	0.086
AIP	0.561 (0.462;0.660)	0.050	0.222	0.435	0.366	0.789	1.731	0.804	0.155
TyG Index	0.573 (0.474;0.673)	0.051	0.140	8.636	0.488	0.649	1.391	0.788	0.137

AUC, Area under the Curve; CI, Confidence Interval; SE, Standard Error; LR, Likelihood Ratio. NLR, Neutrophil Lymphocyte Ratio; PLR, Platelet-Lymphocyte Ratio; MLR, Monocyte Lymphocyte Ratio; NMR, Neutrophil Monocyte Ratio; Lymphocytes/HDL-C, Lymphocyte High Density Lipoprotein Ratio; Monocytes/HDL-C, Monocyte High Density Lipoprotein Ratio; Granulocytes/HDL-C, Granulocyte High Density Lipoprotein Ratio; AIP, Plasma Atherogenic Index; TyG Index, Triglyceride Glucose Index.

The calculated AUC values are as follows: NLR 0.470, PLR 0.490, MLR 0.474, NMR 0.481, LHR 0.567, MHR 0.506, GHR 0.466, AIP 0.561 and TyG index 0.573. NLR, PLR, MLR, NMR and GHR AUC values were below 0.50, indicating that these parameters could not reach sufficient discriminative power in GDM prediction. The AUC values of LHR, MHR, AIP and TyG index were in the range of 0.50–0.60 and these parameters were found to have limited predictive value for GDM prediction.

## 4 Discussion

GDM is a condition that can lead to serious health problems for both mother and baby. These complications can extend from the foetus to the newborn and even into adulthood. Therefore, early detection of GDM is critical to minimise the adverse effects of diabetes. Nowadays, researchers show great interest in identifying parameters that can provide early prediction of GDM. In this diagnostic accuracy study, the predictive value of nine parameters including inflammatory markers (NLR, PLR, MLR, and NMR), HDL-based metabolic inflammation indicators (LHR, MHR, GHR) and metabolic indices (AIP, TyG Index) for the early diagnosis of GDM was examined. The potential of each parameter in the early diagnosis of GDM is discussed in detail under the following sub-headings.

### 4.1 Personal variables

Advanced maternal age and increased BMI are well-established risk factors for GDM as they contribute to insulin resistance and impaired *β*-cell function (40). As observed in our study, the association between high parity and GDM risk is consistent with studies showing that multiparity may contribute to β-cell depletion and impaired glucose metabolism, leading to an increased incidence of GDM (41). Again, consistent with the literature, our study showed that the incidence of GDM increased with advanced maternal age, multiparity and increased BMI (14).

### 4.2 Role of inflammatory markers: NLR, PLR, MLR and NMR

Chronic, moderate inflammation that develops as the maternal immune system adapts during pregnancy plays a critical role in the pathogenesis of GDM. The use of markers such as NLR, PLR, MLR and NMR, which can be easily calculated from a complete blood count, has attracted interest in recent years for the early detection of this inflammatory process. However, studies examining inflammatory markers associated with GDM yield conflicting results. While some studies indicate that these ratios are increased in GDM as an indicator of systemic inflammation, others find no significant difference between the groups or report inconsistent findings. In the light of our findings and the available data in the literature, the possible reasons for these contradictory results are discussed below.

Many studies have reported that NLR and PLR are significantly elevated in GDM as an indicator of systemic inflammation. For example, in a meta-analysis, NLR and PLR were found to be higher in women with GDM compared to the control group, emphasising their potential value as prognostic markers that can be obtained with simple blood tests (42). Similarly, Liu et al. noted that NLR and PLR as well as mean platelet volume were positively associated with GDM risk, suggesting that these markers may be predictive in early pregnancy (43). Wang et al. showed that PLR has clinical value in predicting triglyceride and GDM risk in early pregnancy, supporting the link between inflammatory response and metabolic imbalance (44). These significant findings suggest that NLR and PLR reflect subclinical inflammation and may contribute to the pathophysiology of GDM.

On the other hand, conflicting results in the literature complicate the association of these inflammatory markers with GDM. Aci et al. analysed the relationship between NLR, PLR and systemic immune inflammation index (SII) and GDM risk, emphasised that inflammation data in women were contradictory and reported positive associations in some studies while others reported negative or neutral results (45). In one study, MLR was significant in predicting GDM in the early second trimester, but there was no difference between groups in NMR, PLR and NLR, highlighting the inconsistency in the literature (17). Mertoglu et al. discussed that markers such as NLR may be indicators of subclinical inflammation in women with GDM, pointed out inconsistent findings on the role of NLR in GDM and stated that no relationship could be detected in some subanalyses (46). He discussed that their results differed from other studies and possible reasons for these discrepancies (e.g., population differences or methodological variations). Liu et al. stated that although NLR and PLR are significant, this relationship may weaken in heterogeneous populations (43). Likewise, in a study, it was stated that there were inconsistent results regarding the benefit of NLR in GDM, while some studies stated that NLR increased, in some studies this increase was not observed (47). He stated that the relationship between GDM and NLR may be influenced by factors such as BMI and age. It has been suggested that clinical interpretation of NLR should be done with caution due to the high level of heterogeneity and non-specific nature of NLR, and that conflicting findings may be due to various factors such as statistical power, population heterogeneity and differences in GDM diagnostic criteria. In some studies, although NLR and PLR values were found to be higher in women with GDM, they did not have a statistically significant predictive value, these ratios were not an independent predictor of GDM, but it was emphasised that their role in general inflammation was supported (48, 49).

In our study: NLR, PLR, MLR and NMR evaluated in early pregnancy did not have statistically significant benefits in the prediction of GDM. Our findings in ROC analysis (NLR: 0.470, PLR: 0.490, MLR: 0.474, NMR: 0.481) indicate that these markers are clinically insufficient to predict GDM. There may be more than one reason for the contradictory results in the literature and the negative findings in our study. These reasons reflect the multifactorial nature of GDM and should be considered in the development of early diagnosis strategies.

First, important adaptations in maternal physiology occur during pregnancy; for example, increased immune tolerance may mask fluctuations in inflammatory markers (50). These adaptations may prevent GDM-specific inflammatory responses from becoming sufficiently prominent yet, especially in early pregnancy. In the literature, inflammatory markers have been reported to show more pronounced changes in the second or third trimester of pregnancy (50, 51). This may have led to the failure of markers such as NLR and PLR to reflect the development of GDM in samples obtained in early pregnancy in our study and may partially explain the negative findings. Consequently, the low diagnostic accuracy observed in our cohort strongly suggests that at 10–14 weeks, the systemic inflammatory response to hyperglycemia is not yet sufficiently established to be detected by standard hematological indices, unlike in the second or third trimesters where physiological adaptations are more advanced.

Secondly, methodological heterogeneity between studies - different ethnic groups, GDM diagnostic criteria (e.g., IADPSG vs. Carpenter-Coustan), gestational weeks at which blood samples were taken and methods of analysis - contribute significantly to discrepancies in results (52, 53). For example, the inflammatory response in different ethnic groups is known to be influenced by genetic (e.g., human leukocyte antigen (HLA) variations) and environmental factors (diet, prevalence of obesity) (54, 55). These factors may alter baseline levels of inflammatory markers and their response to GDM, leading to negative results in some cohorts. The high heterogeneity (I^2^ > 50%) observed in meta-analyses also supports this variation (42).

Finally, markers such as NLR, PLR, MLR and NMR are general indicators of inflammation and may not fully reflect the complex mechanism underlying GDM. Instead, more specific biomarkers implicated in the pathophysiology of GDM - for example, cytokines (TNF-*α*, IL-6), adipokines (leptin, adiponectin) or metabolomic panels - may offer higher sensitivity and specificity for early detection (56–58).

In conclusion, our findings and the literature suggest that the role of markers such as NLR, PLR, MLR and NMR in the prediction of GDM has not yet been clarified and that these markers do not behave in the same way in all populations or stages of pregnancy. Our results reinforce the heterogeneity in this field and highlight factors that may limit the clinical use of markers.

### 4.3 Role of HDL-related markers

It is suggested that new markers obtained by the ratio of immune cell counts to HDL-C (LHR, MHR and GHR) can be used to predict metabolic inflammatory disorders of GDM by evaluating both the inflammatory response and the anti-inflammatory potential of HDL together. However, when the literature is examined, it is evident that studies on the effectiveness of these HDL-based parameters for predicting GDM are limited and heterogeneous. While some studies reported that these ratios may be valuable biomarkers for GDM, others did not find a significant relationship or emphasised the limited usefulness of these markers. In the light of the findings obtained in our study and the available data in the literature, the role of these HDL-based inflammatory markers in the prediction of GDM and the possible reasons for the conflicting results are discussed below.

### 4.4 Lymphocyte/HDL-C, monocyte/HDL-C and granulocyte/HDL-C

In recent years, LHR has emerged as a novel inflammatory metabolic marker associated with insulin resistance and is positively associated with pre-diabetes (59, 60). It has been suggested that MHR may be a composite inflammatory biomarker reflecting the balance between inflammation and metabolic status and may be used as a pro-inflammatory indicator in Type 2 Diabetes Mellitus (DM). However, it is not yet clear whether cumulative monocyte/HDL ratio and chronic metabolic inflammation predispose to Type 2 DM (61–63).

Available data indicate that studies on these HDL-based parameters for predicting GDM have been conducted in limited groups and remain very limited and heterogeneous. A study of women with GDM indicated that LHR, MHR and GHR may be valuable biomarkers for predicting GDM. In this study, especially LHR above 3.66 was found to increase the risk of GDM fourfold (16). In other studies, high monocyte/HDL ratio has been accepted as an important marker for GDM. This ratio has been evaluated as an indicator of inflammation and insulin resistance in women with GDM (64–66). In one study, no relationship was found between monocyte/HDL ratio and GDM. It was stated that the usability of monocyte/HDL as an inflammatory marker for GDM is limited and it cannot be used as an inflammatory index for GDM screening (67).

In our study, LHR (AUC 0.567) and MHR (AUC 0.506) had limited effect on the prediction of GDM and GHR (AUC 0.466) had no effect in the analysis of HDL-related inflammatory markers examined in early pregnancy. In addition, no statistically significant results were found between markers between both groups.

There may be several reasons for these contradictory findings in the literature and the results in our study. The important physiological adaptations of pregnancy in maternal metabolism and the immune system, and differences in the composition and functionality of HDL may affect the diagnostic value of conventional HDL-C measurements or HDL-based ratios. As in our study, immunological tolerance and metabolic reprogramming in early pregnancy may prevent GDM-specific inflammatory responses from becoming fully apparent. Moreover, physiological lipid profile variations in the first trimester differ significantly from those in later pregnancy. The functional impairment of HDL and the associated atherogenic shifts often require a longer duration of metabolic stress to develop, which may explain why HDL-based ratios (LHR, MHR, and GHR) did not show significant predictive value at 10–14 weeks.

It is also emphasised in the literature that factors such as different methodologies in the studies, heterogeneity of the populations included, differences in GDM diagnostic criteria and gestational weeks at which blood samples were taken are one of the main reasons for inconsistencies in the results (50, 68). Measurements in early pregnancy may give different results from later gestational weeks when the pathophysiology of GDM becomes more apparent (69).

Finally, these ratios, which are general indicators of inflammation, may not be specific enough to reflect the complex pathophysiology of GDM at an early stage. Advanced biomarkers reflecting more specific molecular pathways involved in the pathophysiology of GDM, such as cytokines or metabolomic panels, may offer higher sensitivity and specificity for early diagnosis (56). Furthermore, individual differences in the biological behaviour of immune cells and HDL, genetic factors, lifestyle and environmental factors may alter inflammatory responses and lipid metabolism from person to person, which may affect the effectiveness of these ratios in predicting GDM (70).

Although studies have expressed HDL-C as an independent risk factor in GDM, they emphasise that its predictive power alone is low, that the predictive effect of HDL-related factors in women with GDM is limited, especially alone, and that it gives more effective results when used in combination with more comprehensive panels or risk models (71–73). The results of our study are in line with this limited effect.

### 4.5 Role of metabolic markers in GDM risk prediction

#### 4.5.1 Atherogenic index

Increased insulin resistance during pregnancy and accompanying dyslipidaemia constitute the main components of GDM pathogenesis. In order to evaluate these lipid metabolism disorders, AIP, which reflects the atherogenic lipid profile, stands out as an innovative marker. The pathophysiological basis of the relationship between AIP and GDM is tried to be explained by mechanisms such as triggering chronic moderate inflammation, increased oxidative stress and endothelial dysfunction. Furthermore, it is suggested that lipid profile changes associated with high AIP values worsen insulin resistance by increasing free fatty acid release and contribute to impaired glucose homeostasis. Although the association between AIP and GDM seems pathophysiologically plausible, the results of the literature are unclear. In the light of the findings obtained in our study and available literature data, the role of AIP in the prediction of GDM is discussed below.

Although the available literature suggests a generally positive association between AIP and GDM, this finding is contradictory. Many studies have reported that high AIP values in early pregnancy increase the risk of GDM and may have a predictive value (53, 58, 74–78). However, although high AIP values were found in some studies, it is stated that the prediction of GDM is limited (73, 78).

In our study, the AUC value of AIP in ROC analysis was calculated as 0.561, which indicates that it may have a limited diagnostic performance in predicting GDM. In addition, there was no statistically significant difference between pregnant women with and without GDM in terms of AIP values (*p* > 0.05). These findings suggest that AIP alone may not be a reliable marker for predicting GDM.

#### 4.5.2 Triglyceride-glucose index

Considering that insulin resistance is one of the main pathophysiological mechanisms of GDM, TyG index, which is thought to be a practical marker reflecting insulin resistance, has been suggested to be a potential marker for predicting GDM. However, studies in the literature contain inconsistent results on the role of the TyG index in GMM prediction. In the light of the findings obtained in our study and the available literature data, the performance of TyG index in the prediction of GDM is discussed below.

In some studies conducted in the first trimester, it has been reported that high TyG index increases the risk of GDM and has a good predictive value (27, 34, 79). However, some studies have shown that its predictive power for GDM remains limited (80–82). Zeng et al. reported that the diagnostic efficiency of the TyG index in GDM was limited (AUC = 0.57), no statistically significant difference was detected between trimesters and TyG was not a powerful early diagnostic tool for GDM. It was also noted that there may be differences between ethnic groups (81). In the study of Liu et al., TyG index was found to be significantly higher than the control group, but no significant association was found with GDM in the first trimester, while significant results were obtained in the second trimester. They emphasised that TyG index may be a potential biomarker for GDM, but there are still inconclusive results about its performance and further research is needed (83).

One of the important factors underlying these inconsistencies in the literature is the significant differences observed in the threshold values of the TyG index between studies. Methodological heterogeneity and lack of standardised thresholds in studies limit the generalisability of this index and its validity in clinical practice. Therefore, it is emphasised that high-quality, standardised studies are needed to prove the diagnostic accuracy of the TyG index (80). According to our current knowledge, the performance of the TyG index in predicting GDM improves when combined with other clinical risk factors (age, BMI, family history), but remains limited when used alone.

In our study, the AUC value of TyG index in ROC analysis was calculated as 0.573, which indicates a limited diagnostic performance in predicting GDM risk. Furthermore, no statistically significant difference was observed between pregnant women with and without GDM in terms of mean values of TyG index (*p* > 0.05). These findings are consistent with studies reporting limited performance in the literature and suggest that TyG index alone may not be a reliable marker for prediction of GDM.

### 4.6 Possible causes of our results regarding metabolic markers

Most of the studies in the literature have important limitations such as methodological differences (different trimester studies, rectospective studies) and population differences. It also has a limited sample size and long-term follow-up data are limited. The presence of confounding factors such as BMI, age, history of GDM, dietary habits and the need for validation studies in different ethnic groups are issues that are constantly emphasised by researchers. There may be many reasons for the contradictory results in the literature and the findings in our study. This may be explained by various methodological (GDM diagnostic criteria, gestational week at which blood samples were taken, sample size), population-based and biological factors. Firstly, it is thought that AIP and TyG tend to increase due to the physiological hypertriglyceridemia expected in advancing gestational weeks. In one study, it was reported that AIP may gradually increase from the first to the third trimester of pregnancy, reaching moderate/high risk categories (84). In many studies where an association was found, the data consisted of lipid values obtained simultaneously with GDM screening (24–28 weeks of pregnancy) (74, 85–88). We believe that the predictive performance of AIP and TyG indices is limited in our study because both physiological insulin resistance and hypertriglyceridemia have not yet developed sufficiently in the early first trimester (10–14 weeks). As noted in the literature, these metabolic maladaptations typically become more pronounced in the second half of pregnancy due to placental hormones; thus, our early sampling window likely corresponds to a latent phase of GDM pathophysiology.

Secondly, lipid metabolism and insulin resistance may vary significantly in different populations. Studies indicate that the effectiveness of metabolic markers in predicting GDM may differ between populations. GDM is known to be more common in racial and ethnic minority groups such as Asian/Pacific Islander, Hispanic and Black women. This suggests that metabolic markers show variable performance in different ethnic groups due to differences in lipid metabolism, insulin resistance patterns and genetic predispositions. For example, a study in Japan found no statistically significant difference in plasma triglycerides and other lipid parameters between women with and without GDM. This result suggests that lipid profile parameters may not be reliable predictors of GDM screening in Japanese women (89). In another study, African Americans were found to have lower TG levels compared to white people, but higher rates of insulin resistance, high blood pressure and fasting glucose. It has been reported that the TG/HDL-C ratio is different in women and shows significant differences between racial/ethnic groups. This suggests that the effectiveness of TG-based indicators as markers of insulin resistance in individuals of African descent is limited (90, 91). These data support that metabolic markers require population-specific validation in different ethnic groups and that race and ethnicity factors must be taken into account when using these markers as predictors. The fact that our study was conducted in the Turkish population is an important factor affecting our results. The dietary habits, lifestyle factors and genetic characteristics of the Turkish population may be different from the previously reported populations in which positive associations were observed. This leads to the important limitation that the results are specific to a particular population and the generalisability to other ethnic groups is limited.

Finally, AIP and TyG indices, which are considered indicators of insulin resistance, may not fully reflect the complex pathophysiology of GDM on their own. GDM is associated with a multifactorial process that includes not only insulin resistance and lipid metabolism disorders but also *β*-cell dysfunction, inflammation, and oxidative stress. Therefore, instead of using AIP and TyG indices alone, their evaluation in combination with clinical risk factors and other metabolic markers (e.g., Hemoglobin A1c (HbA1c), Homeostatic Model Assessment of Insulin Resistance (HOMA-IR)) may provide higher predictive values, as reported in the literature. It should be kept in mind that the clinical manifestation of GDM usually occurs between 24 and 28 weeks of gestation and metabolic disorders become more prominent during this period (92). Therefore, it should be recognized that measurements made early in pregnancy may be insufficient to capture GDM-specific metabolic changes. Therefore, prospective studies with serial measurements across different gestational periods and long-term follow-up are needed to better understand the role of AIP and TyG indices in predicting GDM. When all these factors are evaluated, we think that the AIP and TyG indices alone are insufficient for predicting GDM in early pregnancy. Still, when assessed alongside clinical and metabolic indicators, they may help early detect high-risk cases and plan close glycaemic monitoring with appropriate nutrition/lifestyle counselling. The fact that our study was conducted in early pregnancy is an essential factor in terms of our results.

## 5 Conclusion

### 5.1 Conclusion and future directions

In our study, the effectiveness of easily accessible and cost-effective parameters in predicting GDM in early pregnancy was comprehensively analysed. The main finding of our study was that none of these markers reliably predicted GDM in early pregnancy. The AUC values obtained in ROC analyses indicate that these parameters are clinically limited (LHR, MHR, AIP and TyG) in distinguishing between pregnant women with and without GDM. Our findings emphasise that the use of these biomarkers alone in screening for GDM in early pregnancy may not be appropriate.

### 5.2 The importance of negative findings

We believe that our negative findings and limited predictive values have made an important contribution to the scientific literature. Reporting both negative and positive results in research is critical to strengthening evidence-based medicine practices. Negative results may lead to questioning certain hypotheses or diagnostic approaches and the development of alternative strategies.

### 5.3 Strengths

Our study is the first study in the literature in which 9 parameters (inflammatory markers, HDL-based metabolic inflammation indicators and metabolic indices) were evaluated simultaneously. The strengths of our study include prospective cohort design, standardised GDM diagnostic criteria, collection of blood samples in early pregnancy and simultaneous assessment of multiple biomarkers. In addition, all samples were analysed by standardised methods in the same laboratory, which increases the reliability of our results.

### 5.4 Limitations

This study has several limitations. Firstly, it was adequately powered to detect moderate to large effects but may have been underpowered for very small associations. Observed effect sizes were close to unity, indicating minimal associations; thus, the lack of statistical significance likely reflects negligible effects rather than insufficient power. Secondly, the single-centre nature of the study and the relatively limited sample size may affect the generalisability of the results, and multicentre studies with larger and more diverse populations are needed to confirm our findings. Thirdly, measurements were made at a single time point during early pregnancy, whereas serial measurements could provide a better understanding of dynamic changes in biomarkers and their relationship with GDM. Finally, the markers we evaluated are general indicators of inflammation and metabolic disorders and may not fully capture the complex pathophysiology of GDM; more specific molecular markers, such as cytokines, adipokines, microRNAs, or metabolomic panels, may offer higher specificity and sensitivity for early detection.

### 5.5 Future directions

Our recommendations for future studies are as follows: Multicentre, prospective studies with large samples and including different ethnic groups may clarify the role of these markers in the prediction of GDM. Serial measurements in different trimesters of pregnancy are important to investigate dynamic changes and time-dependent relationships. Furthermore, the development of multivariate risk prediction models in which these biomarkers are evaluated together with clinical risk factors (age, BMI, family history, pre-pregnancy metabolic status, history of GDM) and other biochemical parameters (fasting glucose, HbA1c, HOMA-IR) and the exclusion of confounding factors may provide higher predictive values in the prediction of GDM. Advanced biomarkers that specifically reflect the pathophysiology of GDM (e.g., inflammatory cytokines, adipokines, oxidative stress markers, microRNAs) should be investigated. In the future, integration of multiple biomarkers and clinical data into artificial intelligence and machine learning algorithms may offer new opportunities for early detection of GDM.

### 5.6 Conclusion

The results of our study show that easily accessible inflammatory and metabolic markers in early pregnancy have limited predictive value in the prediction of GDM, but no marker can be used alone with the current data. It also supports that standardised approaches for GDM screening (risk factor-based screening and OGTT at 24–28 weeks) are still the most reliable methods. Our findings emphasise the need to develop more specific and sensitive biomarkers for early detection of GDM and provide an important roadmap for future research. Since early diagnosis and prevention of GDM is critical for maternal and foetal health, it is of great importance to continue research in this field.

## Data Availability

The raw data supporting the conclusions of this article will be made available by the authors, without undue reservation.
